# Infected Urachal Cyst Masquerading as Acute Appendicitis on Point-of-care Ultrasound

**DOI:** 10.5811/cpcem.2022.1.55243

**Published:** 2022-05-05

**Authors:** Victoria Quinn, Francois Luks, Erika Constantine

**Affiliations:** * Brown University Warren Alpert Medical School, Hasbro Children’s Hospital, Department of Pediatrics, Providence, Rhode Island; † Brown University Warren Alpert Medical School, Hasbro Children’s Hospital, Division of Pediatric Surgery, Providence, Rhode Island; ‡ Brown University Warren Alpert Medical School, Hasbro Children’s Hospital, Department of Pediatrics and Pediatric Emergency Medicine, Providence, Rhode Island

**Keywords:** urachal cyst, point-of-care ultrasound (POCUS), appendicitis, case report

## Abstract

**CASE PRESENTATION:**

A seven-year-old male presented to the pediatric emergency department with one day of abdominal pain. His physical exam was significant for rebound, guarding, and tenderness in the right lower quadrant, and his labs demonstrated a leukocytosis. Both a point-of-care ultrasound and radiology-performed ultrasound were concerning for acute appendicitis with a periappendiceal abscess, but on emergent laparoscopy the patient was found to have an infected urachal cyst.

**DISCUSSION:**

Infected urachal remnants are a rare but important cause of pediatric abdominal pain. In this case, inflammation surrounding the patient’s midline urachal cyst triggered a serositis that involved the appendix and pulled the cyst to the right. This created a clinical and radiologic presentation similar to appendicitis. This atypical presentation of an already rare anomaly highlights the importance of maintaining a broad differential during the work-up of pediatric abdominal pain.

## CASE PRESENTATION

**Image 1:**
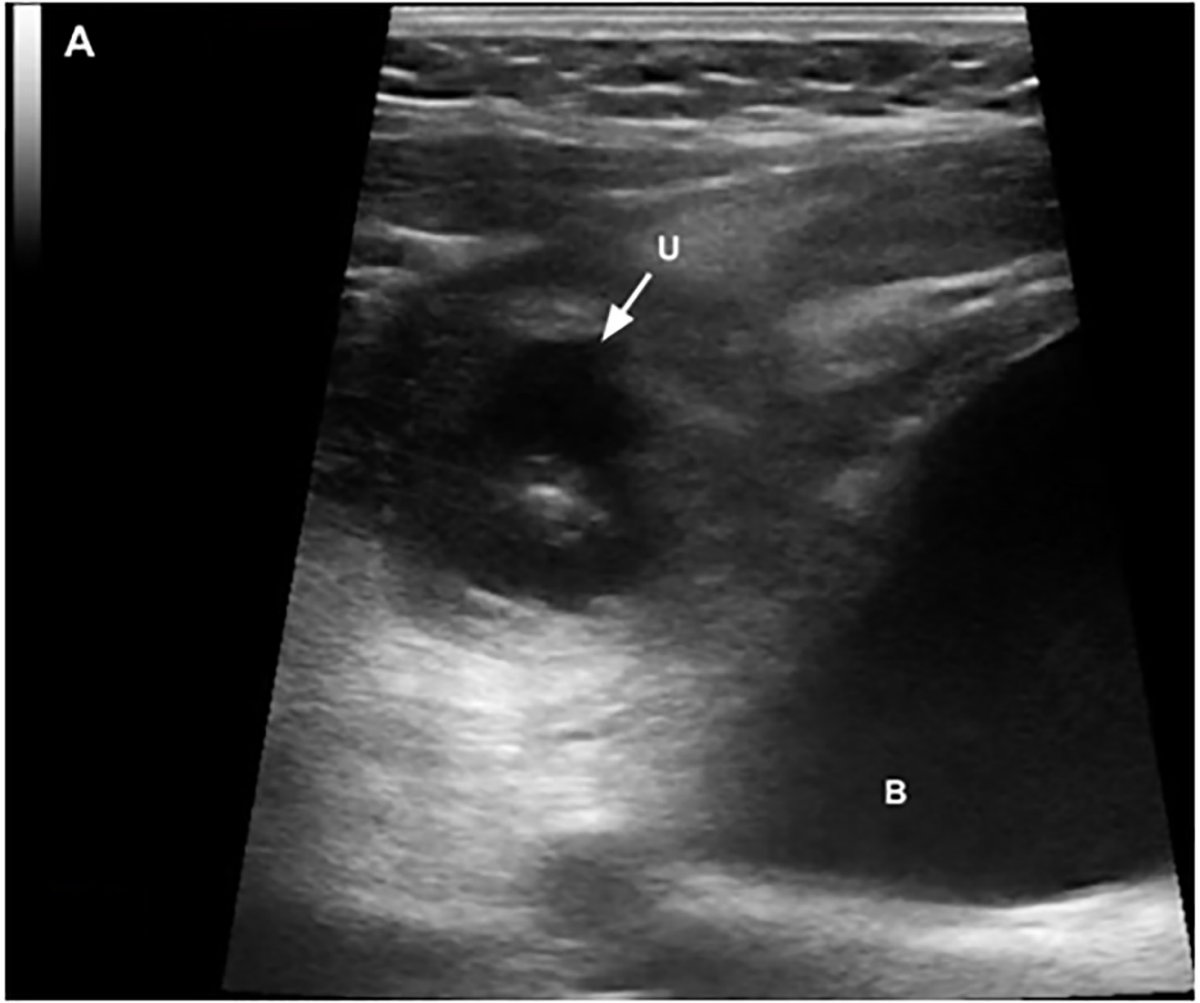
A point-of-care ultrasound image obtained with a linear transducer in the transverse plane illustrates a heterogeneous collection superior and to the right of the urinary bladder surrounded by hyperechoic inflammatory changes. (U = urachal cyst; B = bladder)

**Image 2:**
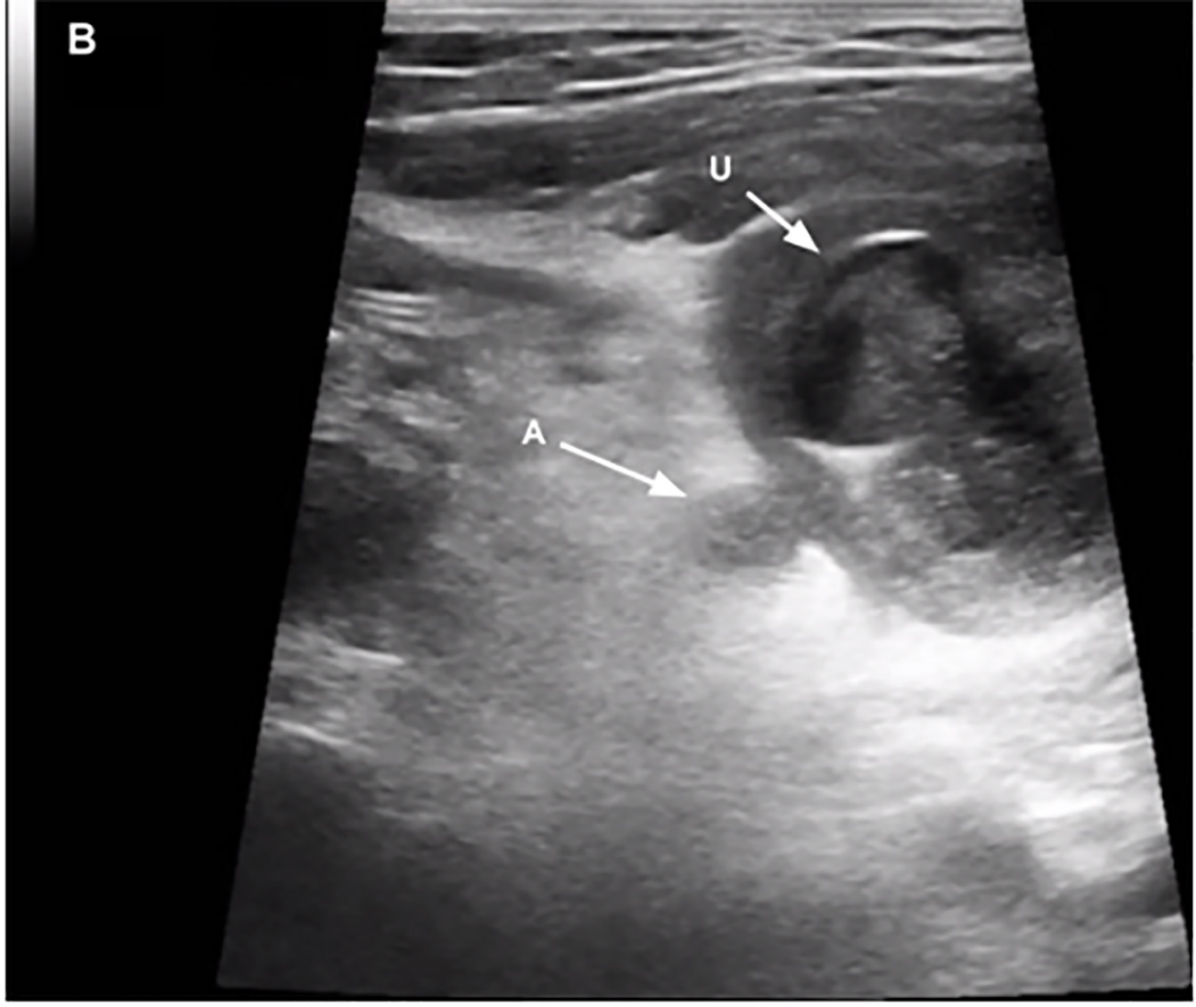
Point-of-care ultrasound image obtained with a linear transducer in the transverse plane illustrating the proximity of the urachal cyst to the distal tip of the appendix. (U = urachal cyst, A = distal tip of the appendix).

A seven-year-old male with no past medical history presented to the pediatric emergency department with one day of right lower abdominal pain. On arrival, he was uncomfortable, but afebrile and hemodynamically stable. Physical exam demonstrated periumbilical and right lower quadrant tenderness with rebound and guarding. Labs revealed a leukocytosis of 17.2 × 10^9^/liter (L) (reference range 4.4 – 11.0 × 10^9^/L) and a negative urinalysis (no leukocytes esterase, nitrites, or blood). Point-of-care and radiology-performed ultrasounds both revealed a well-circumscribed, heterogeneous collection superior and to the right of the bladder, and closely approximated with the distal appendix (Video, Images 1 and 2). These findings, in addition to significant inflammatory changes and posterior acoustic enhancement, were concerning for a perforated appendicitis with periappendiceal abscess. The patient was started on piperacillin/tazobactam and admitted to pediatric surgery.

At laparoscopy, a mildly inflamed appendix without perforation was visualized in the right lower quadrant, but further dissection revealed a significantly inflamed midline mass attached to the superior aspect of the bladder. The surgical team removed the appendix and mass, taking care to suture-ligate its connection with the dome of the bladder. Surgical pathology revealed that the mass was a urothelial-lined cyst consistent with a urachal remnant, with necrosis, hemorrhage, granulation tissue, and acute-on-chronic inflammation. Additionally, the appendix had serosal fibrinopurulent exudate and minimal transmural inflammation. The patient had an uneventful postoperative course.

## DISCUSSION

Urachal remnants arise when the urachus, an embryonic tract between the allantois and the bladder, does not involute. The most common result is a urachal cyst, which forms when either end of the urachus seals off but the middle remains patent, fills with fluid, and can become infected with urologic flora.^1^ Most cysts are asymptomatic and go undiagnosed until they become infected or appear incidentally on imaging. Several case series have highlighted the utility of sonography as a screening modality for uncomplicated urachal remnants, but the diagnostic sensitivity and specificity, especially for infected remnants, remains undefined.^2^ To our knowledge, there is only one other report demonstrating the utility of point-of-care ultrasound to diagnose infected urachal cysts.^3^

**CPC-EM Capsule**

What do we already know about this clinical entity?

*While urachal cysts are the most common urachal remnant, they are still rare and often go undiagnosed until they become infected or appear incidentally on imaging*.

What is the major impact of the image(s)?

*These images of a misdiagnosed urachal cyst demonstrate the variety of sonographic findings associated with infected urachal remnants*.

How might this improve emergency medicine practice?

*This case highlights the importance of imaging structures in multiple planes and maintaining a broad differential of pediatric abdominal pain in the emergency department*.

A urachal cyst typically appears on ultrasound and computed tomography as a non-communicating, fluid-filled pocket between the umbilicus and the bladder. When infected, these cysts are associated with a variety of sonographic and clinical findings based on their size, effect on surrounding tissue, and location of patient pain.^1^,^4^,^5^ While infected urachal cysts typically present with periumbilical pain because they are midline, surrounding inflammation can trigger a serositis that moves the cyst from midline. As demonstrated in this case, the resulting parietal peritonitis caused adhesion of the cyst to the tip of the appendix that pulled the cyst right, presented as right lower quadrant pain, and created sonographic findings that are more typically associated with appendicitis. This case demonstrates the importance of adequately imaging structures in multiple planes, as well as the importance of maintaining a broad differential during the work-up of pediatric abdominal pain to evaluate for common etiologies and rare congenital anomalies.

## Supplementary Information

Video Point-of-care ultrasound performed with a high-frequency linear probe demonstrating a heterogeneous collection superior and to the right of the urinary bladder, closely approximated with the distal tip of the appendix, and surrounded by significant inflammatory change and posterior acoustic enhancement.
